# Retrobulbar/intraconal tube placement in patients with glaucoma: Ahmed FP-7 and tube extender case series with 1 year follow-up

**DOI:** 10.3389/fopht.2025.1408897

**Published:** 2025-02-25

**Authors:** Daniel Laroche, Brian Grodecki, Kara Rickford Grimes, Chester Ng

**Affiliations:** ^1^ Department of Ophthalmology, New York Eye and Ear Infirmary of Mount Sinai Icahn School of Medicine, New York, NY, United States; ^2^ Department of Ophthalmology, Advanced Eyecare of New York, New York, NY, United States; ^3^ Drexel University College of Medicine, Philadelphia, PA, United States; ^4^ New York Medical College, Valhalla, NY, United States

**Keywords:** retrobulbar, intraconal, tube extender, glaucoma, Ahmed FP-7

## Abstract

**Purpose:**

The Ahmed FP-7 valve is useful in the management of refractory glaucoma. However, this can often have ocular hypertensive phase and subconjunctival fibrosis that can lead to increased medication use and failure. We report how retrobulbar and intraconal plate placement with tube extension can avoid the ocular hypertensive phase, lower intraocular pressure, and reduce medication burden.

**Patients and methods:**

This is a retrospective case series of 4 patients with glaucoma who underwent Ahmed FP-7 valve and retrobulbar/intraconal tube placement with a one-year follow-up.

**Results:**

One-year results in 4 patients with advanced glaucoma and pseudophakia revealed a pre-operative intraocular pressure of 21 mmHg on 5.5 medications. At one year, the post-operative intraocular pressure was 10.25 on 0 medications. The vision and visual fields were stable. One patient required drainage of a choroidal effusion and one patient required burping of viscoelastic on post-operative day one.

**Conclusions:**

The combined insertion of the Ahmed FP-7 valve and silicone tube inserted into the retrobulbar/intraconal space has been shown to prevent ocular hypertensive phase, lower intraocular pressure, and reduce medication burden at one year.

## Introduction

Glaucoma is a well-known cause of irreversible blindness (1). Despite efforts to detect glaucoma early, many patients will present with advanced glaucoma. Although early intervention with eye medications, selective laser trabeculoplasty, early cataract extraction, and microinvasive glaucoma surgery benefit many patients, untreated individuals can progress to advanced glaucoma (2, 3). Glaucoma tube shunts are increasingly used for advanced glaucoma patients due to potential complications with trabeculectomy with mitomycin blebs (4).

The Ahmed FP-7 valve is well known to have an ocular hypertensive phase that can often lead to increased medication use and failure (5). Retrobulbar/intraconal tube shunts have been shown to lower intraocular pressure (IOP) and not have ocular hypertensive phases (6). Retrobulbar tube placement allows aqueous fluid to bypass the subconjunctival space, avoid ocular hypertensive phase, and prevent subconjunctival scarring by diverting aqueous fluid to the retrobulbar/intraconal space (7). This pilot study aims to report the efficacy and safety of this novel retrobulbar/intraconal technique after one year.

## Materials and methods

We report a case series of four patients with advanced glaucoma that underwent retrobulbar/intraconal placement of the Ahmed FP-7 valve (New World Medical, Inc, Rancho Cucamonga, CA, US) and silicone tube extension (Tube extender, New World Medical, Inc, Rancho Cucamonga, CA, US). This retrospective single-center analysis covers a one-year follow-up period for each patient. The measured parameters included intraocular pressure (IOP), number of medications, visual field mean deviation, and adverse events. Optical coherence tomography (OCT) results were included, if available. Written consent to publish each case has been obtained. This report does not contain any personal identifying information.

In this case series, in preparation for the procedure, the eye was sterilely prepped with povidone-iodine and draped while a lid speculum was placed. A double-armed 8-0 vicryl suture was then passed through the superior corneal stroma to retract the globe downward, and a superotemporal conjunctival peritomy was performed. The Ahmed FP-7 valve was primed using a 27-gauge cannula, injecting ∼1 cc of balanced salt solution (BSS). The functionality of the implant was confirmed by allowing the BSS to flow through the plate. A 10–0 prolene suture was used to attach the tip of the silicone tubing (300-micrometer lumen from tube extender, New World Medical) to the silicone plate just behind the valve, using a 3–1–1 knot. Then, the tube extender was tied near the exit of the Ahmed FP-7 valve with a 10-0 prolene suture with a 3-1-1 knot. Notably, the tube extender was not inserted in the valve opening but was secured just posterior to it.

**Figure 1:**
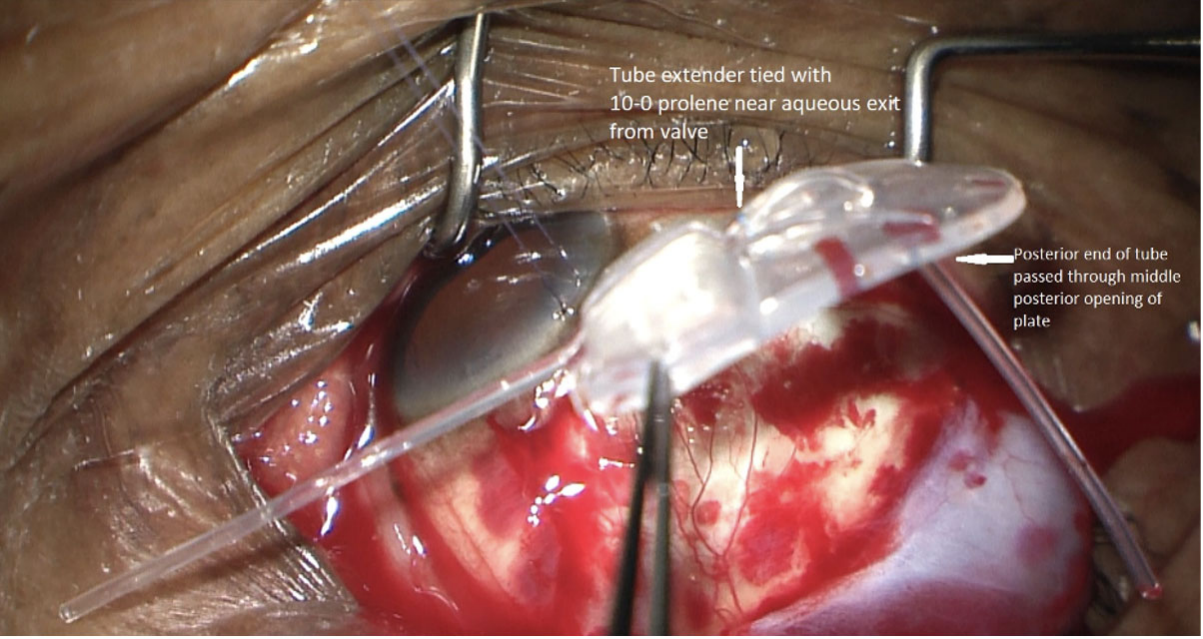
Ahmed valve and additional silicone tubing from tube extender tied with 10-0 prolene.

**Figure 2:**
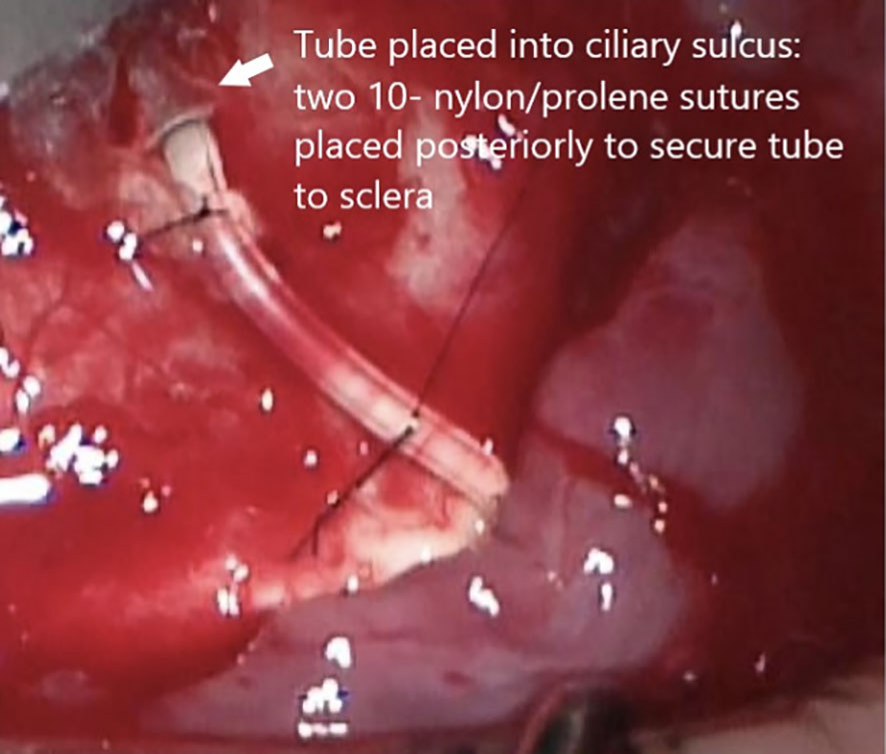
Suturing of tube to sclera near ciliary sulcus entry and as posterior as possible.

The posterior end of the silicone tube was then passed through the central posterior hole of the Ahmed FP-7 plate to direct the tube posteriorly into the retrobulbar/intraconal space (**Figure 1**). The combined Ahmed FP-7 valve and retrobulbar silicone tube addition were inserted into the superotemporal quadrant as far posteriorly as possible within the retrobulbar/intraconal space. The plate, along with the silicone tube extension, was left floating in the intraconal space. The anterior tip of the full length of the tube attached to the Ahmed FP-7 valve was cut at a 5-degree bevel posteriorly to prevent iris obstruction into the ciliary sulcus. The bevel was the only portion of the tube that was cut. Using calipers, a distance of 1.5 mm was measured from the corneal limbus superotemporally. A 23-gauge needle was used to create an entry point into the ciliary sulcus superotemporally. The tube of the Ahmed FP7 plate was inserted into the sulcus behind the iris and anterior to the intraocular lens, positioned just outside of the pupillary axis. The tube was then secured to the sclera with a 10-0 nylon suture in a 3-1-1 knot configuration just posterior to its insertion to the eye. A second 10-0 nylon suture was tied through the tube and sclera approximately 10 mm posteriorly to the limbus, also using a 3-1-1 knot (**Figure 2**).

A 4x8 mm corneal scleral patch (Katena) was cut using Stevens scissors, placed over the anterior tube of the Ahmed FP-7 valve, and secured with four interrupted 8-0 vicryl sutures. The conjunctiva was pulled anteriorly to cover the scleral patch, and closure was completed with two separate running locking 8-0 vicryl sutures. Finally, intracameral diluted moxifloxacin was administered, followed by a subconjunctival injection of triamcinolone. Post-operatively, all glaucoma medications were discontinued in the surgical eye, and a post-operative drop regimen of prednisolone acetate four times a day (QID), ketorolac three times a day (TID), and ofloxacin QID was initiated.

## Case reports

### Case 1

A 59-year-old white female with a history of osteogenesis imperfecta, glaucoma, and dry eye presented with complaints of difficulty with keeping up with taking several eye drops. Her past surgical history was significant for cataract extraction of both eyes, as well as a Goniotomy and intrascleral suprachoroidal microtube performed 4 years earlier in the right eye. Her past medical history was significant for hypertension. Her current medications were amlodipine 10 mg po QID, brinzolamide 1 drop OU BID, timolol/brimonidine OU BID, netarsudil/latanoprost OU QHS, and pilocarpine 2% OD TID. Ocular examination revealed a best corrected visual acuity (BCVA) of 20/25 OD and 20/20 OS. There was an afferent pupillary defect present in the right eye. Intraocular pressures (IOPs) were 10 mmHg OD and 11 mmHg OS. Examination of the eyelids revealed punctual plugs present in the inferior puncta OU. There was inferior conjunctival scarring OD due to previous ciliary sulcus intrascleral suprachoroidal tube surgery with a well-healed corneal patch. Both corneas were clear and posterior chamber intraocular lenses (PCIOLs) were present OU. A suprachoroidal tube was seen on dilated ocular examination in the ciliary sulcus inferotemporally OD. The cup-to-disc ratios were 0.95 OD and 0.85 OS with diffuse thinning of the neuroretinal rim OU. The visual fields were OD VFI 67% MD -14.17 and OS VFI 99% MD -5.16. Central corneal thickness (CCT) was 410 um OD and 409 um OS. The patient agreed to undergo Ahmed FP-7 valve with retrobulbar/intraconal tube placement to lower her IOP and decrease the number of medications for the right eye.

At post-op day number 1, the BCVA was 20/200 OD and 20/80 OS. The superotemporal sclera had a corneal patch beneath the conjunctiva. There was a tube in the ciliary sulcus superotemporally in the right eye. The IOP was 5 mmHg OD and 13 mmHg OS. Glaucoma medications were discontinued in the right eye and the patient took prednisolone acetate and ofloxacin QID OD. One week later, her BCVA was 20/40 in the right eye and 20/20 in the left eye. The IOPs were 15 mmHg OD and 18 mmHg OS. She was instructed to discontinue the ofloxacin and taper the prednisolone acetate. At post-op week 3, her BCVA was 20/60 OD and 20/20 OS. The IOPs were 10 mmHg OD and 12 mmHg OS. At post-op week 4, her BCVA was 20/25 OD and 20/20 OS. The IOPs were 13 mmHg on no medications in the right eye and 13 mmHg on the continued glaucoma medications in the left eye. At 6 months post-op, her BCVA was 20/25 right eye and 20/20 left eye. The IOPs were 15 mmHg OD and 14 mmHg OS. At 12 months post-op, the BCVA was 20/25 OD and 20/20 OS and the IOPs were 12 mmHg OU. She was on no medications in the right eye and taking brinzolamide OS BID, brimonidine/timolol OS BID, and netarsudil/latanoprost OS QHS. Both her cup-to-disc and VF were stable. The visual fields were OD VFI 71% MD -12 and OS VFI 96% MD -4.

### Case 2

An 82-year-old Black female with glaucoma and progressively worsening vision on several medications came for further evaluation. She had hypertension, and her past surgical history was significant for cataract extraction OU and Ahmed FP-7 valve insertion OD. Her ocular medications were netarsudil/latanoprost OS QHS, dorzolamide-timolol OS, BID pilocarpine 2% OS QID, and acetazolamide 500 mg (OS) QHS. On ocular examination, her BCVA was no light perception (NLP) OD and 20/25 OS. There was an afferent papillary defect and monocular sensory exotropia in the right eye. Examination of the conjunctiva revealed a superior corneal patch in the right eye with an Ahmed valve bleb visible. The pupil was miotic in the left eye. The IOPs were 31 mmHg OD and 21 mmHg OS. There were well-positioned intraocular lenses and a tube present in the ciliary sulcus of the right eye superotemporally. The cup-to-disc ratio was 0.95 OU. The corneal pachymetry of the left eye was 501 um. The VF of the left eye revealed a VFI of 25% and an MD of -23.31. The patient agreed to undergo Ahmed FP-7 valve with retrobulbar tube placement in the left eye to lower the IOP and reduce the number of medications and the risk of visual field progression.

On post-op day 1, the BCVA in the left eye was 20/50 and the IOP was 7 mmHg OS. The patient was taking prednisolone acetate OS QID and ofloxacin OS QID. On post-op day 7, the vision deteriorated to hand motions (HM) in the left eye and the IOP was 3 mmHg. The anterior chamber was shallow in the left eye and retinal examination revealed large choroidal effusions. Viscoelastic was injected into the anterior chamber with a 25-gauge needle after a betadine prep, local anesthesia, and informed consent. The anterior chamber deepened, however, the choroidal effusions and hypotony persisted. One week later, the patient underwent drainage of choroidal effusions with two inferior sclerotomies in each quadrant and reformation of the anterior chamber. On post-op day 1, the vision improved to counting fingers (CF) in the left eye and the IOP was 10 mmHg. On post-op day 7, the vision improved to 20/400 and the IOP was 6 mmHg in the left eye. By post-op week 3, the vision improved to 20/70 and the IOP was 7 mmHg in the left eye. At 3 months post-op, the vision improved back to its baseline of 20/50 with an IOP of 10 mmHg in the left eye. At 12 months follow-up, the vision was stable at 20/50 left eye and the IOP was 7 mmHg with no ocular medications. The visual field improved to VFI 45% MD -18.61 OS.

### Case 3

A 68-year-old Indian woman with diabetes, hypertension, end-stage renal disease (ESRD), and glaucoma was referred for uncontrolled intraocular pressures. Her past surgical history was significant for cataract extraction OU. Her medications included amlodipine, metformin, insulin, and dorzolamide-timolol OU BID. Ocular examination revealed a BCVA of HM in the right eye and CF in the left eye. The IOPs were 53 mmHg in the right eye and 24 mmHg in the left eye. There was mild corneal edema OU and diffuse corneal scarring OD. Gonioscopy revealed synechial angle closure OD and Schaffer grade IV angle open OS. Slit-lamp examination revealed a well-positioned PCIOL OU. The cup-to-disc ratio was 0.99 in the right eye - with a significant loss of the neuroretinal rim - and 0.5 in the left eye. Retinal examination revealed clinically significant macular edema (CSME) OS and nonproliferative diabetic retinopathy OU. A paracentesis was performed in the right eye and acetazolamide 500 mg extended release was given. The IOP decreased to 31 mmHg and 21 mmHg two hours later. The patient agreed to an Ahmed FP-7 valve with retrobulbar tube insertion in the right eye and underwent the procedure without any complications.

**Figure 3:**
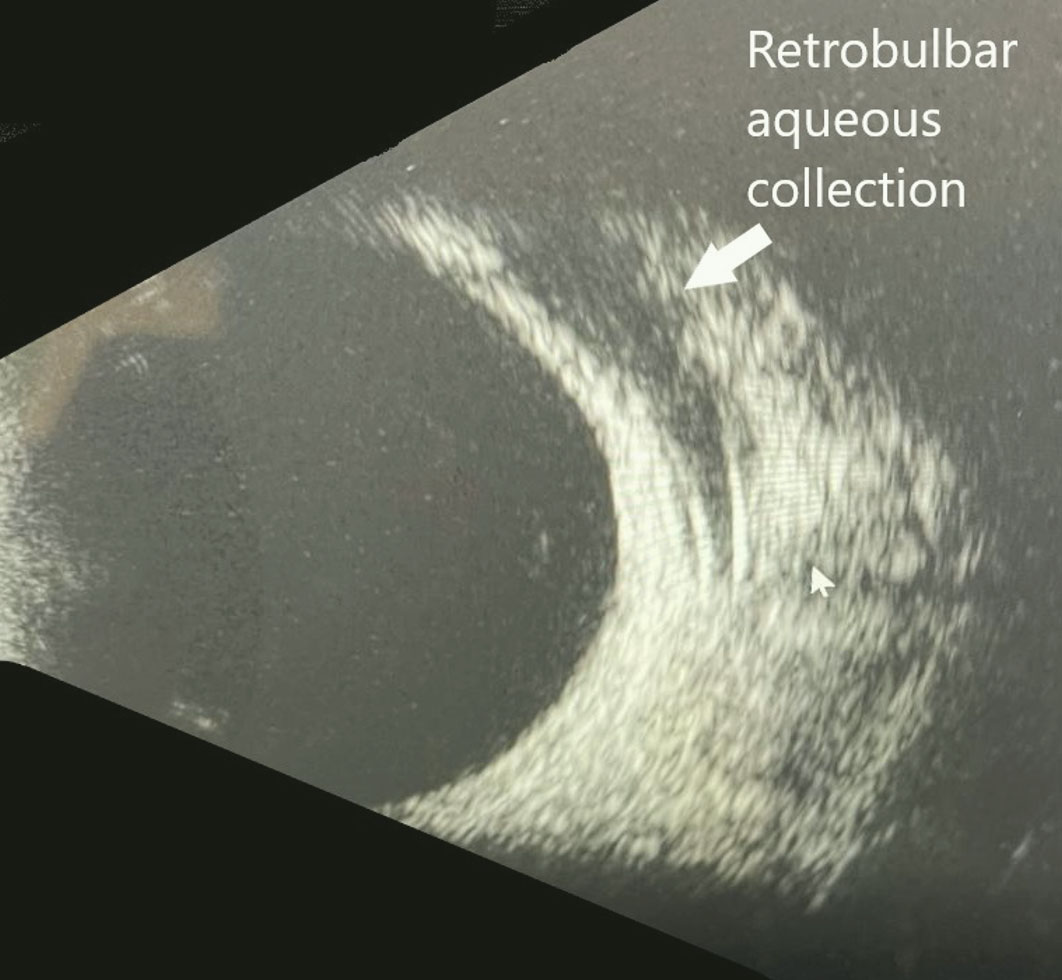
B-Scan showing aqueous collection in retrobulbar space.

On post-op day one, the vision was CF in the right eye and 20/200 in the left eye. The IOP was 79 mmHg OD and 34 mmHg OS. There was retained viscoelastic in the right eye. The patient underwent paracentesis to “burp” viscoelastic via the wound to lower the IOP to 7 mmHg. The patient had stopped medications in the left eye and was instructed to resume dorzolamide-timolol BID, netarsudil/latanoprost QHS, and brimonidine OS BID in the left eye. At one week post-op, the BCVA was CF OD and 20/200 OS, while the IOPs were 20 mmHg OD and 19 mmHg OS. At 1 month post-op, the BCVA was CF in the right eye and 20/140 in the left eye and the IOPs were 16 mmHg OD and 13 mmHg OS. Ofloxacin was discontinued and prednisolone acetate was tapered. The patient was instructed to continue dorzolamide-timolol OU, netarsudil/latanoprost OU, and acetazolamide 500 mg sequel OS. The patient was evaluated by a retinal specialist and underwent a focal laser for CMSE in the left eye. At post-op 5 months, the BCVA was CF OD (**Figure 3**, B-scan of the right eye) and 20/140 OS. The IOP was 18 mmHg OD and 27 mmHg OS. The patient agreed to an Ahmed FP-7 valve with retrobulbar tube insertion in the left eye to lower IOP and reduce the number of medications.

On post-op day 1, the BCVA was 20/400 OD and CF OS. The IOP was 16 mmHg OD and 4 mmHg OS. There were superotemporal corneal patches and well-positioned tubes in the ciliary sulcus OU. The patient was instructed to discontinue all glaucoma medications and use prednisolone acetate OS QID and ofloxacin OS QID. The post-op BCVA at 1 week was 20/400 OD and 20/100 OS. The IOPs were 14 mmHg OD and 22 mmHg OS. Brinzolamide/brimonidine was added OS BID. At the post-op 3-week visit, the BCVA was CF OD and 20/140 OS. The IOP was 11 mmHg OD and 14 mmHg OS. At 6 months and 12 months post-op, the vision remained the same. At 6 months post-op, the IOP was 11 mmHg OD and 16 mmHg OS. At 12 months, the IOP was 10 mmHg OD and 20 mmHg OS, while only taking brinzolamide/brimonidine in the left eye. Visual fields were not reliable due to the patient’s diabetic retinopathy.

### Case 4

A 53-year-old Black female with glaucoma and hypertension presented for a follow-up visit. The patient reported being intolerant to pilocarpine and acetazolamide. Her past surgical history was significant for cataract extraction OU and trabeculectomy OU. Her medications were Genteal tears, amlodipine 5 mg, losartan 50 mg, brimonidine.2% OS BID, dorzolamide 2% OS BID, and latanoprost 0.005 OS QHS. The patient also had an allergy to beta-blockers. Her BCVA was 20/80 in the right eye and 20/25 in the left eye. The IOPs were 24 mmHg OD and 15 mmHg OS. There were superior conjunctival blebs OU and the corneal epithelium had mild superficial punctuate keratopathy in both eyes. Gonioscopy revealed Schaffer grade IV open angles OU and well-positioned PCIOLs in both eyes. The cup-to-disc ratios were 0.95 OU with very thin neuroretinal rims and advanced glaucomatous cupping. HVF MD was -33.56 OD and -30.82 OS with advanced loss. The target IOP in the right eye was 9-12 mmHg. Due to the elevated intraocular pressure and intolerance to medications, the patient agreed to undergo Ahmed FP-7 valve with retrobulbar tube insertion in the right eye to lower the IOP and reduce the number of medications. The patient tolerated the procedure well without any complications.

**Figure 4:**
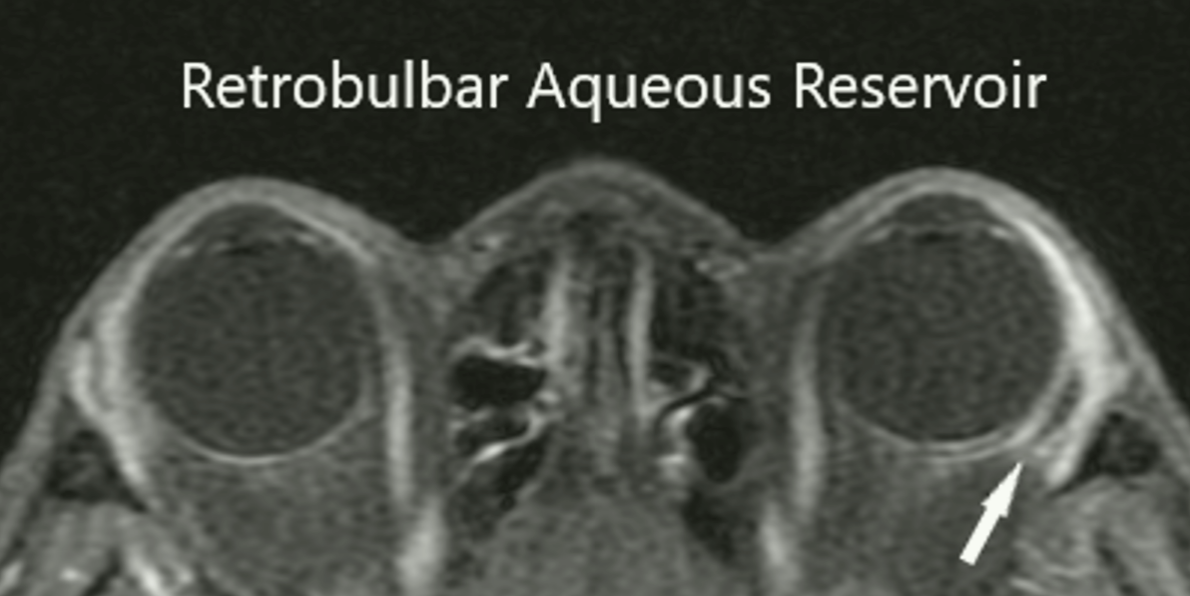
MRI showing aqueous in retrobulbar space.

**Figure 5:**
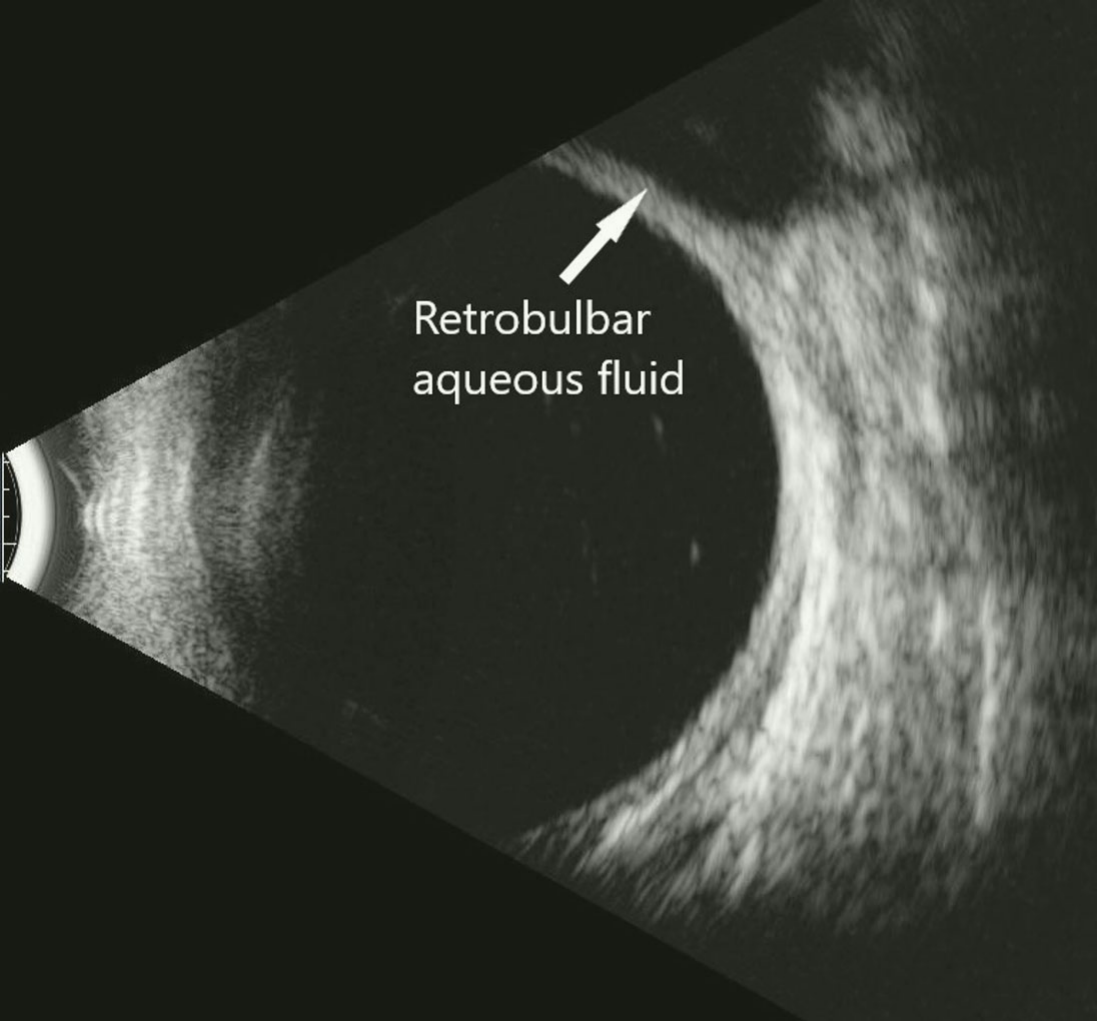
B-scan of aqueous in retrobulbar space.

On post-op day 1, the patient’s ocular medications were prednisolone acetate OD QID, ofloxacin OD QID, brimonidine 0.2% OS BID, dorzolamide 2% OS BID, and latanoprost 0.005% OS QHS. The vision was HM in the right eye and 20/25 in the left eye. The IOPs were 3 mmHg OD and 15 mmHg OS. The anterior chamber was shallow with peripheral choroidal effusions in the right eye. At post-op week 1, the BCVA was HM OD and 20/25 OS, while the IOP was 5 mmHg OD and 14 mmHg OS with decreased shallowing of the anterior chamber in the right eye. The ofloxacin OD was discontinued at this time. At the post-op 1 month visit, the vision improved in the right eye to 20/200 and the IOP increased to 11 mmHg in the right eye and 16 mmHg in the left eye. At the 4-month post-op visit, the vision improved to the baseline of 20/70 OD and the IOP was 8 mmHg OD and 16 mmHg OS. The VF MD was -33.6 OD and -.12 OS. At post-op month 12, the vision was stable at 20/80 OD and the IOP was 7 mmHg OD and 16 mmHg OS. The patient was on no glaucoma medications for her right eye (**Figure 4**, MRI of orbit right eye; **Figure 5**, B-scan of aqueous in the right eye).

## Discussion

In patients with advanced glaucoma, the Ahmed FP-7 valve with retrobulbar/intraconal tube extension and placement can avoid ocular hypertensive phase, prevent subconjunctival scarring, lower intraocular pressure, and reduce medication burden. With this procedure, patients do not have a bleb, reducing the risk of persistent leaks and other related complications (2, 8). Placement of the plate in the retrobulbar/intraconal space creates a more posterior aqueous reservoir. The aqueous humor can then be absorbed posteriorly by lymphatics around the optic nerve and within the retrobulbar/intraconal space. Additionally, the plate and tube float posteriorly in the retrobulbar space and no impingement of the optic nerve was noted (**Figure 4**). In small eyes/orbits or during nasal placement, the sides of the Ahmed FP-7 valve can be trimmed without affecting the posterior direction to fit in the small space.

Results in 4 patients with advanced glaucoma and pseudophakia at baseline demonstrated a pre-operative IOP of 21 mmHg on 5.5 medications. At one year post-op, the average IOP was reduced to 10.25 mmHg (P=0.114), and the IOP was reduced in all three patients with an elevated pre-operative IOP. Postoperatively, IOP-lowering medication use was significantly reduced to 0 in all patients (P=0.0031). The vision and visual fields were stable. One patient required drainage of a choroidal effusion, and one patient required burping of viscoelastic on post-op day one.

Performing this procedure on pseudophakic patients reduces the risk of adverse effects of choroidal expansion due to increased volume in the eye from the absence of the lens (9). All patients had the tube placed in the ciliary sulcus, reducing the risk of corneal edema and failure from placement of the tube in the anterior chamber (10). Although in Case 3 the left eye underwent the procedure, we did not include it since we did not have 1 year follow-up.

Retrobulbar/intraconal tube shunts can be an effective way to control intraocular pressure in patients with advanced glaucoma. It is important to ensure that the retrobulbar/intraconal position is beyond the equator to prevent anterior subconjunctival scarring that can lead to failure, as seen in traditional subconjunctival tubes.

The efficacy of retrobulbar shunts was first demonstrated in their use to rehabilitate fibrotic shunt devices. The fibrotic blebs, which impede aqueous fluid flow, are salvaged by way of a retrobulbar tube extension that resolves the sequestration of the aqueous fluid (11). Because Ahmed valves are a valved system, the risk of hypotony is reduced but can still occur from peritubular outflow or device malfunction. This is also why we chose this valve, as its position in the retrobulbar/intraconal space will reduce the risk of hypotony. Should a bleb still be formed with anterior placement of the Ahmed valve plate, if well-positioned, the presence of the tube extender near the valve opening allows aqueous fluid to egress further to the retrobulbar/intraconal space, reducing the risk of filtration failure.

Given that traditional plate shunt devices and retrobulbar tubes have different mechanisms of action, we have shown that placing the plate in the retrobulbar/intraconal space may have a superior effect. This can potentially limit the number of future interventions, as fibrosis of the bleb would not need further management since the retrobulbar extension tube is already in place. As such, it may be beneficial to combine shunt devices and retrobulbar/intraconal tubes as an initial intervention in patients with advanced glaucoma. The four cases we reported in this paper demonstrate the promise of this combined procedure in controlling the IOP and lowering medication burden (**Table 1**). We are currently performing this procedure in lieu of traditional trabeculectomy and glaucoma subconjunctival tube shunts due to the excellent results we are seeing. Limitations of this pilot study include the small sample size and short duration of follow-up. Further research is required with a larger number of patients and longer follow-ups.

**Table 1:** Patient demographics and mean pre-operative and post-operative intraocular pressures and medications.

1 year data	Age	Sex	Medical History	Ocular Surgical History	Ocular Findings	Side of Surgery	IOP		Medications		VF MD		Post-Op Findings
							Pre-Op	Post-Op	Pre-Op	Post-Op	Pre-Op	Post-Op	
Case 1	59	F	Osteogenesis imperfecta	Cataract extraction OU, goniotomy OD, intrascleral suprachoroidal microtube OD	Dry eye OU, afferent pupillary defect OD, inferior conjunctival scarring OD, punctal plugs OU	OD	10	12	5	0	-14.17	-12	Corneal patch of superotemporal sclera beneath conjunctiva
Case 2	82	F	Hypertension	Cataract extraction OU, Ahmed FP-7 valve insertion OD	Afferent pupillary defect OD, monocular sensory exotropia OD, superior corneal patch OD with Ahmed valve bleb, miosis OS	OS	21	12	6	0	-23.31	-18.61	Shallow anterior chamber, large choroidal effusions, hypotony
Case 3	68	F	Hypertension, type 2 diabetes, end-stage renal disease	Cataract extraction OU	Nonproliferative diabetic retinopathy OU, mild corneal edema OU, diffuse corneal scarring OD, clinically significant macular edema OU	OD	31	10	7	0	N/A	N/A	Retained viscoelastic
Case 4	53	F	Hypertension	Cataract extraction OU, trabeculectomy OU	Superficial conjunctival blebs OU, superficial punctate keratopathy OU	OD	22	7	4	0	-33.6	-33.6	Shallow anterior chamber, peripheral choroidal effusions

IOP, Intraocular pressure; VF MD, Visual field mean deviation.

## Data Availability

The original contributions presented in the study are included in the article/Supplementary Material. Further inquiries can be directed to the corresponding author.
